# Rapid Detection Methods for Bacterial Pathogens in Ambient Waters at the Point of Sample Collection: A Brief Review

**DOI:** 10.1093/cid/ciaa498

**Published:** 2020-07-09

**Authors:** Jing Li, Yanzhe Zhu, Xunyi Wu, Michael R Hoffmann

**Affiliations:** Linde + Robinson Laboratories, California Institute of Technology, Pasadena, California, USA

**Keywords:** waterborne pathogens, exposure risk assessment, detection methods, rapidity, portability

## Abstract

The world is currently facing a serious health burden of waterborne diseases, including diarrhea, gastrointestinal diseases, and systemic illnesses. The control of these infectious diseases ultimately depends on the access to safe drinking water, properly managed sanitation, and hygiene practices. Therefore, ultrasensitive, rapid, and specific monitoring platforms for bacterial pathogens in ambient waters at the point of sample collection are urgently needed. We conducted a literature review on state-of-the-art research of rapid in-field aquatic bacteria detection methods, including cell-based methods, nucleic acid amplification detection methods, and biosensors. The detection performance, the advantages, and the disadvantages of the technologies are critically discussed. We envision that promising monitoring approaches should be automated, real-time, and target-multiplexed, thus allowing comprehensive evaluation of exposure risks attributable to waterborne pathogens and even emerging microbial contaminants such as antibiotic resistance genes, which leads to better protection of public health.

Access to adequate water, sanitation, and hygiene (WASH) has long been a significant public health concern and an international development policy. According to the World Health Organization, global mortality attributable to waterborne diseases is estimated to be > 2.2 million per year, among which about 1.4 million are children, resulting in nearly $12 billion per year of economic loss worldwide [1]. It is estimated that diarrhea alone amounts to 842 000 deaths per year due to unsafe WASH and includes 361 000 deaths of children < 5 years of age, mostly in low-income countries [2]. Ultrasensitive, rapid, and specific monitoring platforms for bacterial pathogens in ambient waters at the point of sample collection are essential for timely water quality surveillance and microbial risk assessment. Therefore, the development of such platforms plays a key role in predicting and assessing the risk of disease outbreaks and providing quality care in healthcare settings such as improving the effectiveness of vaccine distribution.

Microbial detection techniques are usually classified into phenotypic methods and molecular methods. Culture-based methods as the mainstream of phenotyping have the advantages of cost-effectiveness and simplicity, and remain the gold standard for bacterial monitoring and identification. However, it requires days for culture-based methods to provide conclusive results, which greatly hampers their applications in water quality monitoring [3]. Molecular analyses including conventional polymerase chain reaction (PCR)–based methods, immunology-based methods, etc, however, require lengthy processes of sample pretreatment (eg, concentration, cell lysis, purification), expensive equipment, and trained personnel in centralized laboratory facilities. The demanding requirements of molecular methods represent a major disadvantage for their application in resource-limited communities [4, 5]. In addition, the majority of currently available molecular techniques have low precision (~20%) and are poorly suited for absolute quantification, thus having limited application in low-concentration pathogen detection [6]. To tackle this problem, in addition to enhancing specificity and mitigating competitive side reactions, researchers have also been exploring the “digital detection” concept. It realizes absolute quantification through separating the sample into sufficient partitions followed by individual molecular reaction and endpoint counting of positive and negative signals in each reaction [6, 7]. In addition, biosensor is also a promising technique for future waterborne pathogen monitoring systems. Biosensor generally provides more reliable results from real-time measurements and allows rapid analysis without the requirement of complicated pretreatment steps such as the target enrichment process, which still has a lot of room to be developed [8–10].

Overall, microbial pathogen detection is urged to be ultrasensitive, rapid, simple, low-cost, field-deployable, and easily operable by undertrained individuals for applications in environmental surveillance. Over the past years, numerous research advances have been made in such integrated platform for detection and identification of bacterial pathogens including but not limited to *Salmonella enterica* serovar Typhi (*S.* typhi) in water. Here, we review representative technologies categorized into cell-based methods, nucleic acid amplification methods, and biosensors. We also further discuss the needs of future developments on microbial monitoring platforms in the underdeveloped parts of the world.

## CELL-BASED DETECTION METHODS

Compared to molecular-based detection platforms that target specific nucleic acids or proteins, cell-based detection methods offer direct identification and measurement with relative simple workflows [11]. Utilization of commercial instruments simplifies the construction of cell-based detection platforms. Recent development of miniaturized analysis systems has further promoted the efficiency and portability of cell-based detection methods, thus enabling complex diagnostics or monitoring procedures.

Miniaturized cell cultivation techniques based on microfluidic devices and Lab-on-a-Chip technologies consume less fluid, take less volume, and usually have higher tolerance toward ambient conditions, thus reducing the total cost and time for bacterial analysis [12]. One example of miniaturized cell cultivation is a palm-size device developed by Futai et al utilizing Braille display, monolithic surface, modified culture media and transparent heater [13]. This device was successfully used to culture highly carbon dioxide (CO_2_)–dependent cells in nonpreferable growing environment with limited CO_2_, humidity and a non-37°C temperature. Even for uncultivatable microbial species in various environments, an isolation chip with miniature diffusion chambers was developed to achieve parallel cultivation and isolation [14]. However, these miniaturized cell cultivation routines unavoidably take a long time, can usually be labor intensive, and require skilled operators.

Compared to cell culture, flow cytometers (FCMs) for direct cell counting enable fast quantification of the total bacterial community in the environment with high reproducibility and relatively small standard deviation. More importantly, many commercial FCMs are available for adaptations and the setup of FCM is suitable for automation, making FCM a great candidate for online routine bacterial monitoring [15]. Besmer et al used an automated in situ FCM analysis platform to help characterize the temporal variation of dynamic aquatic environments enabled by a commercial FCM (C6 flow cytometer, BD Accuri, San Jose California) coupled with a fully automated staining robot [16, 17]. Going one step further, Props et al combined the use of real-time FCM and advanced fingerprinting metrics, which aided the detection and characterization of microbial dynamic changes with a high temporal resolution of 10–30 seconds [18]. Nevertheless, FCM techniques have some major drawbacks, including difficulties in distinguishing between live and dead cells and specific strains of bacteria, and in discriminating bacterial aggregates and clusters. Incorporating microscopic imaging to FCM could boost the specificity of this detection platform. For example, an automatic imaging FCM was developed with a deep learning–based phase-recovery and holographic-reconstruction framework to generate pictures of micro-object in water samples without fluorescence triggering, and the pictures generated could be used for characterization [19]. However, current holograms taken by the microscopy and reconstructed images do not have a resolution high enough for specific bacterial pathogen characterization and thus further research is needed.

Besides miniature cell cultivation and FCM, other online cell-based sensing methods have also been developed. A real-time sensor using multiangle light scattering (MALS) technology was developed by Sherchan et al. By comparing the light scattering patterns after using a laser beam to strike particulates in water (including organic particles and microbial cells) with light scattering patterns in the computerized database, data obtained was characterized and the load of injected *Escherichia coli* was back-calculated [20]. Due to the existence of fluorophores in bacterial cells such as tryptophan, phenylalanine, or nucleic acids, which emit fluorescence light after excited by ultraviolet light, Simões and Dong developed an optical microfluidic sensor based on tryptophan intrinsic fluorescence with 3D-printing prototyping [21]. Furthermore, direct 3D image recognition for online pathogen detection was enabled by the combination of a sample-holding flow cell and a field imaging system (including a light source, a magnifying lens, and a camera). An image analysis system was developed to analyze 59 parameters of the images obtained and was able to distinguish between bacteria and abiotic particles. 3D image recognition analysis also provides quantification results, which correlates well with actual bacterial counts [22]. Tables 1 and 2 summarize specific detection parameters and comments on the application and detection parameters of the above-mentioned cell-based technologies.

**Table 1.** Pathogen Detection Methods and Their Samples Studied

Detection Method			Phenotypic or Genetic	Waterborne Microbial Agent Tested	Complex Sample Matrices Tested	Treated Volume, mL
A. Cell-based	A1. Isolation chip		Phenotypic	Total bacteria	Seawater and soil	NA
	A2. Online flow cytometry		Phenotypic	Total bacteria	Drinking water, river water, and groundwater	0.015
	A3. Real-time flow cytometry		Phenotypic	Total bacteria	Nonchlorinated municipal drinking water, river water, and pond water	0.016/min
	A4. MALS sensor		Phenotypic	*Escherichia coli*	Distilled and tap water	600
	A5. Optical microfluidic sensor based on tryptophan intrinsic fluorescence		Phenotypic	*E. coli* and *Legionella*	Distilled water	NA
	A6. Novel optical sensor		Phenotypic	Total particles	Nonchlorinated water and water from cattle slaughterhouse	200
B. NAA	PCR-based	B1. Coaxial channel-based DNA extraction and microfluidic PCR	Genetic	*E. coli*	Milk	10
	LAMP-based	B2. Self-contained microfluidic gLAMP	Genetic	*Proteus hauseri*	Serum	NA
				*Vibrio parahaemolyticus*		
				*Salmonella* subsp *enterica*		
				*E. coli*		
		B3. Centrifugal microfluidic automatic wireless endpoint LAMP	Genetic	*E. coli*	Chicken meat	NA
				*Salmonella* spp		
				*Vibrio cholerae*		
		B4. One-step single-layer membrane for digital LAMP	Genetic	*E. coli*	Culture media	NA
				*Salmonella* Typhi		
				*Enterococcus faecalis*		
		B5. Asymmetric double-layer membrane for digital LAMP	Genetic	*E. coli*	Unprocessed environmental water	10
				*Salmonella* Typhi		
		B6. In-gel LAMP	Genetic	MS2	Culture media	NA
C. Biosensor	C1. MOF-bacteriophage biosensor		Phenotypic	*Staphylococcus aureus*	Pastry cream	0.6
	C2. Impedimetric paper-based biosensor		Phenotypic	Cultures from sewage sludge	Synthetic wastewater	NA
	C3. Immunomagnetic separation and colorimetric paper-based device		Phenotypic	*Salmonella* Typhimurium	Bird feces and whole milk	1
	C4. Real-time amperometric immunoassay amplified by nanomaterial		Phenotypic	*E. coli*	Water	0.2
	C5. Phage-mediated separation with quantitative PCR detection		Combined	*E. coli* O157:H7	Agricultural water and city water	1
	C6. Carbon nanotube multilayer biosensors and on-chip LAMP		Combined	*E. coli* O157:H7	Juice and milk	1

Abbreviations: LAMP, loop-mediated isothermal amplification; MALS, multiangle light scattering; MOF, metal-organic framework; NA, not available; NAA, nucleic acid analysis; PCR, polymerase chain reaction.

**Table 2.** Pathogen Detection Methods and Their Technical Characteristics

Detection Method	Limit of Detection	Recovery Efficiency, %	Dynamic Range	Time to Answer, h	Absolute or Relative Quantification	Trained Personnel Required	Tests at Species Level	Ready for Field Test	Reference
A1	NA	Up to 50%	~500 cells	2 wk	Relative	Yes	No	No	[14]
A2	10^3^ cells/mL^−1^	NA	10^3^–10^6^ cells/mL^−1^	0.25	Absolute	No	No	Yes	[16, 17]
A3	10^3^ cells/mL^−1^	NA	~10^3^ cells/mL^−1^	0.25	Absolute	No	No	Yes	[18]
A4	10^3^ CFU/mL^−1^	NA	10^3^–10^6^ CFU/mL^−1^	0	Relative	Yes	No	No	[20]
A5	1.4 × 10^3^ CFU/mL^−1^	NA	7 × 10^5^ to 1 × 10^4^ CFU/mL^−1^	0	Relative	Yes	No	Yes	[21]
A6	1.6 × 10^2^ particles/mL^−1^	NA	1.6 × 10^2^–5 × 10^6^ particles/mL^−1^	10	Relative	No	No	Yes	[22]
B1	12 CFU/mL^−1^	97.4–100.6	NA	1.5	Relative	No	Yes	No	[23]
B2	3 copies/μL^−1^	NA	3–3000 copies/μL^−1^	1.2	Relative	No	Yes	Yes	[24]
	3 copies/μL^−1^		3–3000 copies/μL^−1^						
	2 copies/μL^−1^		2–2000 copies/μL^−1^						
	3 copies/μL^−1^		3–3000 copies/μL^−1^						
B3	3 × 10^−5^ ng/μL−1 or 2.7 × 10^4^ CFU/mL^−1^	NA	3 × 10^−5^–3 × 100 ng/μL^−1^	1	Relative	No	Yes	Yes	[25]
B4	11 copies/μL^−1^	NA	11–1.1 × 10^5^ copies/μL^−1^	1	Absolute	No	Yes	Yes	[26]
B5	0.3 cells/mL^−1^	99.9	0.3–10 000 cells/mL^−1^	1	Absolute	No	Yes	Yes	[27]
	3 cells/mL^−1^	NA	3–10 000 cells/mL^−1^						
B6	0.7 PFU per reaction	NA	1–1000 PFU per reaction	0.5	Absolute	No	Yes	Yes	[28]
C1	31 CFU/mL^−1^	96–104	40–4 × 10^8^ CFU/mL^−1^	0.33	Relative	No	Yes	Yes	[29]
C2	1.9 × 10^3^ CFU/mL^−1^	NA	10^3^–10^6^ CFU/mL^−1^	0.75	Relative	Yes	No	Yes	[30]
C3	10^2^ CFU/mL^−1^	8.84–21.3	NA	1.5	Relative	Yes	Yes	Yes	[31]
C4	50 CFU/mL^−1^	NA	50–10^7^ CFU/mL^−1^	0.53	Relative	No	Yes	Yes	[32]
C5	10^2^ CFU/mL^−1^	45.4–80.2	10^2^–10^6^ CFU/mL^−1^	2	Relative	Yes	Yes	Yes	[33]
C6	1 CFU/mL^−1^	101–112.1	5–10^5^ CFU/mL^−1^	2	Relative	Yes	Yes	Yes	[34]

Abbreviations: CFU, colony-forming units; NA, not available; PFU, plaque-forming units.

Many methods mentioned in the section have been successfully implanted for days or even months with full automation, and can be constructed easily with commercial instruments. However, the sensitivity of these methods can be easily influenced by different environmental factors and the detection limit is relatively high. Moreover, it is challenging to identify specific pathogens solely based on cell-level analysis, not to mention their genetic information. Therefore, further molecular level detections are needed to secure higher sensitivity and specificity.

## NUCLEIC ACID AMPLIFICATION DETECTION PLATFORMS

Compared to phenotyping methods, molecular methods typically based on the quantification and identification of specific genomic segments of the pathogen’s genomes allow rapid, highly specific, and more sensitive detection, which better fit the expectations of timely monitoring and effective surveillance of aquatic pathogens in a range of water environmental settings. In this section, advances in monitoring methods based on PCR and loop-mediated isothermal amplification (LAMP) are respectively discussed.

## PCR-BASED METHODS

The major drawback of PCR-related methods usually lies in their long response time and limited portability, since they rely on fussy thermal cycling and require additional equipment to detect the amplification products [35]. Another drawback is that trained personnel with experimental skills are needed to perform the assays, thus making the PCR-based systems impractical in resource-limited settings [4, 5]. Therefore, there is an urgent demand for a mobile and automated PCR-based device to monitor water microbial quality. Microfluidics have been demonstrated to provide a higher surface-to-volume ratio and a higher rate of mass and heat transfer, thus offering better performance than conventional systems due to significantly reduced reaction time [36]. Zhang et al reported a microfluidic PCR system integrated with the sample pretreatment technique of coaxial channel-based DNA extraction that was able to detect *E. coli* in milk matrix [23]. Detailed information about this system can be found in Tables 1 and 2. Some companies have directly tackled the mobility issue of PCR systems by developing handheld PCR instruments as shown in Table 3. Nguyen et al investigated the feasibility of using the Biomeme handheld quantitative PCR (qPCR) system for rapid (< 50 minute) on-site detection and monitoring of *Flavobacterium psychrophilum* in filtered water samples [37]. The study showed a close match between the results of the Biomeme handheld qPCR system and those of traditional bench qPCR, highlighting the feasibility of field-based qPCR systems in rapidly detecting and timely monitoring bacterial pathogens in water.

**Table 3.** Summary of Commercially Available Handheld Quantitative Polymerase Chain Reaction Systems

Company	Item	Weight, kg	Footprint, cm^2^
Chai	Open quantitative PCR^a^	4	28.0 × 24.0
Ubiquitome	Freedom 4^b^	Not available	10.2 × 20.3
Ubiquitome	Liberty 16^c^	3.2	21.2 × 11.0
Amplyus	miniPCR^b^	0.45	12.7 × 5.1
Biomeme	Franklin^b^	0.91	About the size of a soda can

Abbreviation: PCR, polymerase chain reaction.

^a^Product information is from https://www.chaibio.com/openqpcr.

^b^Product information is from Reference 35.

^c^Product information is from https://insights.ubiquitomebio.com/liberty16-personal-qpcr- machine.

## LAMP-BASED METHODS

LAMP is one of the most commonly used isothermal amplification methods [23, 38] and has attracted the most attention due to its high specificity, high amounts of amplification product, and superior tolerance to inhibitors [39]. Moreover, LAMP can be carried out at a constant temperature, so that it does not require a thermal cycler, which simplifies the detection procedure and allows better portability compared to PCR-based methods. Chen et al introduced a self-contained microdevice to in-gel LAMP (gLAMP) for multiplexed pathogen detection in complex clinical samples such as serum [24]. *Escherichia coli*, *Proteus hauseri*, *Vibrio parahemolyticus*, and *Salmonella* subspecies were simultaneously detected with high selectivity and sensitivity, as shown in Tables 1 and 2. Another major merit of the detection system was that the microchip preloaded with agarose solution containing LAMP reagents could maintain activity for 30 days when stored at 4°C, allowing the long-term storage and transportation of LAMP reagents, which is essential for LAMP-based point-of-use applications [24]. Sayad et al developed a wireless automatic endpoint detection system using centrifugal microfluidics for food safety examination. Foodborne pathogenic bacteria including *E. coli*, *Salmonella* species, and *Vibrio cholerae* in chicken meat were successfully detected with the sample-to-response time of < 1 hour [25]. Moreover, since this system is performed in an entirely automated way with the help of Bluetooth wireless technology, it is accessible for field application in environmental water samples. However, for the methods described above, the adaptability to environmental water matrix rather than food or blood needs further investigation and validation; in addition, the above methods were semiquantitative and not suitable for absolute quantification. Hoffmann’s laboratory has done a lot of work on developing rapid microbial pathogen detection systems based on digital LAMP (dLAMP) for absolute quantification in environmental waters [26–28]. Lin et al demonstrated that 1-step LAMP can be successfully performed on single-layer commercial polycarbonate membrane to achieve absolute quantification of the genome DNA of *E. coli*, *Enterococcus faecalis*, and *S.* Typhi [26]. Lin et al further reported the development and validation of the simpler and more robust double-layer membrane for dLAMP of bacterial pathogens in complex environmental waters. Absolute quantification of *E. coli* and *S.* Typhi spiked in unprocessed pond water and seawater could be completed within 1 hour with the sensitivity down to single cell [27]. Huang et al developed a gLAMP system enabling absolute quantification of microbial pathogens in environmental waters within 30 minutes at a very low cost of $5 per test. Bacteria (*E. coli* and *S.* Typhi) and viruses (bacteriophage MS2) were immobilized with LAMP reagents in polyethylene glycol hydrogel matrix and were then amplified [28]. Although the authors demonstrated that the above system could also be used for absolute quantification of bacterial targets including *E. coli* and *S.* Typhi, relevant detection limits were not reported, which needs further validation. More detailed information about all of the above-mentioned LAMP-based systems can be found in Tables 1 and 2. In this emerging field, a range of rapid and easy-to-operate platforms have been developed for low-concentration pathogen detection. It has great potential for future application in point-of-sample detection in field upon proper modification of consumables such as reagents and microchips.

## BIOSENSORS

**Figure 1.**
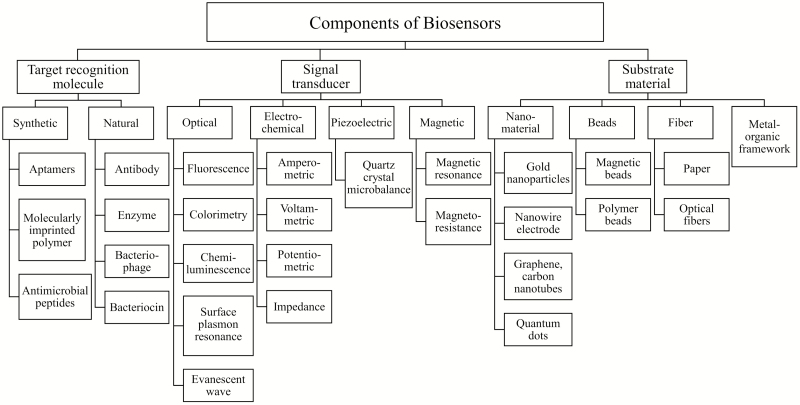
Recent developments in biosensors for bacterial pathogen detection. Widely used or innovative target recognition molecules, signal transducers, and substrate materials are summarized based on Justino et al [40], Kumar et al [41], and Vikesland and Wigginton [42].

Biosensors are analytical devices that consist of target recognition molecules and signal transducers to detect the interaction between the recognition molecules and the specific target. Innovations in recognition molecules and signal transduction methods, as summarized in Figure 1, are emerging to achieve sensitive, rapid, and specific pathogen detection. We note that thorough reviews are available on various types of recognition molecules and signal transducers applicable to waterborne bacterial pathogen detection [40, 41]. Below we highlight novel biosensors that are portable for in-field applications or hold promise for online water quality monitoring.

The novel combination of target recognition molecules and new substrates for their immobilization has been demonstrated to boost the sensitivity of biosensors and the applicability in-field applicability. For example, Bhardwaj et al conjugated bacteriophage onto metal-organic framework (MOF) for specific quantification of *Staphylococcus aureus* [29]. The MOF, NH_2_-MIL-53(Fe), functioned as a water-dispersible and stable matrix, and also as an optical transducer whose reduction in photoluminescence was proportional to target bacterial concentration. This type of stable and economical biosensor with notable quantification performance could be an attractive solution to scale up for point-of-sample-collection detection. However, it should be noted that such target-specific bacteriophage is not available for every bacterial pathogen. As an alternative class of recognition molecules, aptamers (synthetic single-stranded oligonucleotides) can fold into designed 3D structure to bind specific targets. The sequence of the aptamers can be selected in vitro through systematic evolution of ligands by exponential enrichment, and the easily synthesized aptamers have high stability, specificity, and affinity to the targets [43].

Integration of nanomaterials with paper microfluidics has led to development of convenient portable biosensor devices. Commercial test strips, such as RapidCheK and Watersafe, are available for environmental detection of *E. coli* and *Salmonella* Typhimurium. However, these commercial kits mainly use colorimetric detection based on nanoparticle aggregation caused by antibody–antigen reaction, which takes hours to give qualitative results [44]. Using an alternative detection approach, Rengaraj et al conjugated concanavalin A, the recognition molecule binding saccharide on bacterial cell surfaces, onto commercial hydrophobic paper with screen-printed conductive carbon ink for impedance measurement. This device has potential in-field applicability in terms of portable instrumentation and relative assay stability against environmental disturbance [30]. However, as typical to capillary force-driven paper microfluidics, the sample size at microliters is too small to be relevant for environmental pathogen monitoring without a prior sample concentration step. To overcome this limitation, Srisa-Art et al adopted an approach combining immunomagnetic separation using anti-*Salmonella* coated Dynabeads and paper-based sandwich immunoassay using the detection enzyme β-galactosidase, which forms a red-violet product with chlorophenol red galactopyranoside for colorimetric detection. The immunomagnetic separation enabled species-specific capture and enrichment from a 1-mL sample [31]. Although the detection device is paper-based, laboratory equipment such as vortex and pipette was still required for the immunomagnetic separation step. To adapt paper microfluidics for in-field environmental detection, the integration of pathogen-specific separation with biosensors represents both an opportunity and a challenge.

For automated and low-cost bacterial pathogen monitoring, immunoassay-based electrochemical biosensors are approaching commercialization, owing to the consistent assay performance and easily automated instrumentation [45]. For example, based on an electrochemical biosensor, Altintas et al developed a fully automated portable system for real-time amperometric measurements of *E. coli*–specific immunoassay on a microfluidic chip [32]. The instrument prototype with programmed fluid manipulation, electrochemical measurements, and user interface was also developed and tested, thus showing great promise for commercialization. However, since the protein-based recognition reaction is intrinsically weak and susceptible to matrix effect, the majority of these novel biosensors are still limited in sensitivity and specificity compared to nucleic acid analysis (NAA) methods. One solution would be to employ biosensors for target capture utilizing the specific target recognition, while using a nucleic acid–based method to amplify target DNA or RNA for detection. Wang et al demonstrated this approach with bacteriophage-coated Dynabeads for magnetic separation of pathogenic *E. coli* followed by qPCR detection of total bacterial DNA [33]. Li et al combined antibody-coated carbon nanotube multilayer biosensors for specific capture of *E. coli* and microfluidic chip-based LAMP detection [34]. The latter study achieved single cell detection in 1 mL complex samples such as juice and milk [33, 34]. More detailed information on above biosensors can be found in Tables 1 and 2. With the automated platforms available for LAMP and PCR, these studies demonstrated that coupled biosensor-NAA would be a promising approach for further development of a fully automated environmental pathogen detection system.

## CONCLUSIONS 

Portable systems for rapid, ultrasensitive, and specific environmental pathogen monitoring are essential in risk assessment, outbreak prevention, and vaccine distribution for low-resource settings. Recent advances in cell-based, nucleic acid–based, and biosensor-based platforms are reviewed here, with a focus on promising solutions for bacterial pathogen detection in ambient waters at the point of sample collection. Among the reviewed technologies, miniaturized PCR instruments is the most well-developed and commercialized method that is readily deployable in field for sensitive and specific pathogenic bacterial detection, as summarized in Table 3. For biosensors, the combination of biosensor and NAA-based detection holds promise for improved detection efficiency and thus deserves further research and commercial development. Overall, future research should focus on Lab-on-a-Chip pretreatment approaches that can be integrated with subsequent detection [46], entirely automated devices with preloaded reagents, multiplex detection systems, and online real-time monitoring. Such platforms would benefit further comprehensive and timely hazard identification, exposure risk assessment, and pollution control and management. For example, to cope with the global health crisis caused by widespread and fast-evolving antibiotic resistance genes (ARGs), point-of-sample-collection gene sequencing [47] has been developed. This technique provides information on hundreds of ARG subtypes and toxin genes for a range of water environments. Acquiring this information in-field is a pressing need not only for pathogen source tracking, but also for preventing ARG dissemination across various environments.

## Notes

***Financial support.*** This work was supported by the Bill & Melinda Gates Foundation (grant number OPP1111252 and OPP1192379).

***Supplement*** ***sponsorship.***This supplement is funded with support from the Coalition against Typhoid Secretariat, housed at the Sabin Vaccine Institute in Washington, DC and made possible by a grant from the Bill & Melinda Gates Foundation.

***Potential conflicts of interest.*** The authors: No reported conflicts of interest. All authors have submitted the ICMJE Form for Disclosure of Potential Conflicts of Interest.
